# Complete plastome sequence of *Reevesia pycnantha* Y. Ling (Malvaceae): a rare medicinal tree species in South Asia

**DOI:** 10.1080/23802359.2022.2032439

**Published:** 2022-02-03

**Authors:** Zhang Yu-Han, Chen Pei-Dong, Da-Juan Chen, Xiu-Rong Ke, Wang Hua-Feng

**Affiliations:** Hainan Key Laboratory for Sustainable Utilization of Tropical Bioresources, College of Tropical Crops, Hainan University, Haikou, China

**Keywords:** *Reevesia pycnantha* Y. Ling 1951, Malvaceae, plastome, genome structure

## Abstract

Here, we report and characterize the complete plastome of *Reevesia pycnantha,* Y. Ling 1951, which is a rare tree in the plant family Malvaceae. It is distributed in central and southern Jiangxi, Fujian, Guangdong, Hunan, and other provinces of China, where it is endemic. It grows in subtropical climates at middle and low altitudes of 200–800 meters within valleys, along mountain foothills, or on hillsides, in evergreen broad-leaved forests or at forest edges. Our results show that the length of the complete plastome is 161,964 bp, including 129 genes consisted of 81 protein-coding genes, 37 tRNA genes and 8 rRNA genes. It exhibits the typical quadripartite structure and gene content of angiosperms plastomes and comprises two inverted repeat (IRS) regions of 2,469 bp, a large single copy (LSC) region of 90,657 bp, and a small single-copy (SSC) region of 20,315 bp. The total G/C content in the plastome of *R. pycnantha*,Y. Ling 1951 is 36.8%. The complete plastome sequence of *R. pycnantha,* Y. Ling 1951will make contributions to the conservation genetics of this species as well as to phylogenetic studies in Malvaceae.

## Introduction

*Reevesia pycnantha,* Y. Ling 1951 is a rare tree in the plant family Malvaceae that is endemic to China and occurs within central and southern Jiangxi, Fujian, Guangdong, Hunan and other places. Its habitat has a subtropical climate, in which it grows at middle and low altitudes of 200–800 meters within valleys, along mountain foothills, or on hillsides, within evergreen broad-leaved forests or at forest margins. In China, *R. pycnantha,* Y. Ling 1951 is utilized medicinally; in particular, its fruits, flowers, leaves and bark are used to treat blood circulation disorders and rheumatism (Li et al. 2008). To support improved utilization of this species, sustainability, and conservation, we report the complete plastome of *R. pycnantha,* Y. Ling 1951, and we expect that this will also support future phylogenetic investigations of Malvaceae.

In this study, we sampled the fresh leaves of *R. pycnantha,* Y. Ling 1951 from Hangzhou Botanical Garden (Hangzhou, 120°07′ E,30°15′ N) and keep them in silica gel. Total genomic DNA was extracted from dried leaf tissue using the cetyltrimethyl ammonium bromide (CTAB) protocol of Doyle and Doyle (1987). From the collection, both a voucher specimen (H.-F. Wang, A116) and extracted DNA was deposited in the Herbarium of the Institute of Tropical Agriculture and Forestry (HUTB), Hainan University, Haikou, China.

We obtained clean plastome sequence data following the protocol in Zhu et al. (2018), and assembled it in GetOrganelle v1.7.5.0. For the assembly, we set the maximum number of extensions 15, assessed k-mer values of 21, 55, 85, 115, and set the organellar type to embryophyta. We performed annotations according to *Reevesia botingensis,* H. H. Hsue 1963 (NC_054169.1) in Geneious Prime v2021.1.1 (Biomatters Ltd, Auckland, New Zealand) and applied corrections using Sequin (2016).

Our results show that the plastome of *R. pycnantha,* Y.  Ling 1951 bears the typical quadripartite structure of angiosperms and is a total of 161,964 bp in length. It consists of two Inverted Repeats (IRs) of 25,496 bp each, a Large Single Copy (LSC) region of 90,657 bp, and a Small Single-Copy (SSC) region of 20,315 bp. The plastome contains 129 genes, comprising 81 protein-coded genes (five of which are duplicated in the IRs), 37 tRNA genes (seven of which are duplicated in the IRs), and 8 rRNA genes (5S rRNA, 4.5S rRNA, 23S rRNA, and 16S rRNA) (four of which are duplicated in the IRs). The total G/C content of the plastome of *R. pycnantha,* Y. Ling 1951 was 36.8%, and the G/C content of the LSC, SSC and IR regions was 34.6, 31.5 and 42.9%, respectively.

**Figure 1.**
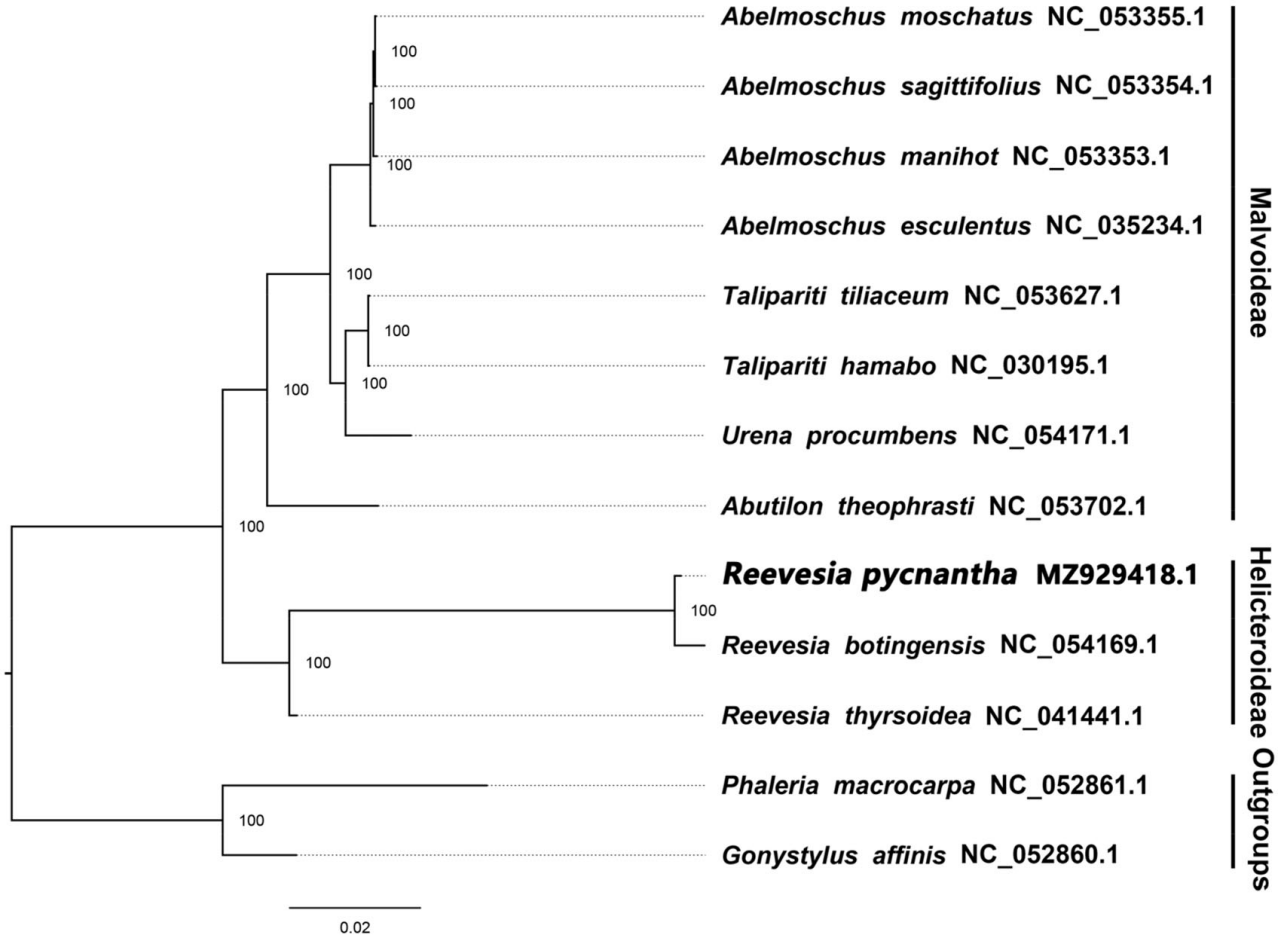
The ML phylogeny of 13 complete plastome sequences of Malvales including the newly sequenced *R*eevesia *pycnantha,* Y. Ling 1951. Numbers adjacent to nodes represent bootstrap support values and numbers right of terminal taxon names represent GenBank accession numbers. To the far right, subfamily affiliation within Malvaceae is shown.

We used the plastome of *R. pycnantha,* Y. Ling 1951 along with twelve other complete, published plastomes of Malvaceae, including three species of *Reevesia*, to reconstruct a maximum likelihood (ML) phylogeny in RAxML (Stamatakis 2006) using its implementation on CIPRES (https://www.phylo.org/). In RAxML, we set the model of nucleotide substitution to GTR + G and performed 1,000 bootstraps replicates using *Phaleria macrocarpa,* (Scheff.) Boerl. 1900, NC_052861.1 (Thymelaeaceae) and *Gonystylus affinis,* Radlk. 1886, NC_052860.1 of Thymelaeaceae as the outgroup. Our phylogeny revealed that *R. pycnantha,* Y. Ling 1951 is closer to *R. botingensis,* H. H. Hsue 1963 and *R. thyrsoidea,* Lindl 1827 than to other species of Malvoideae (Figure 1). Within the phylogeny, the majority of nodes were highly supported.

The plastome presented here may serve as a genomic resource to support conservation and sustainable utilization efforts for this rare species and future evolutionary and ecological investigation of Malvaceae and Malvales.

## Data Availability

The genome sequence data supporting the results of this study were deposited in GenBank of NCBI (https://www.ncbi.nlm.nih.gov/) with accession number MZ929418. The associated BioProject, SRA, and Bio-Sample numbers are PRJNA748537, SRR15498083 and SAMN20607949, respectively. A specimen was deposited at HUTB (http://sweetgum.nybg.org/science/ih/herbarium-details/?irn=124686), Hainan University (https://ha.hainanu.edu.cn/home2020/, H.-F. Wang and hfwang@hainanu.edu.cn) under the voucher number H.-F. Wang, A116.
